# Serological and molecular prevalence of selected canine vector borne pathogens in blood donor candidates, clinically healthy volunteers, and stray dogs in North Carolina

**DOI:** 10.1186/1756-3305-7-116

**Published:** 2014-03-24

**Authors:** Nandhakumar Balakrishnan, Sarah Musulin, Mrudula Varanat, Julie M Bradley, Edward B Breitschwerdt

**Affiliations:** 1 Intracellular Pathogens Research Laboratory, Center for Comparative Medicine and Translational Research, Raleigh, NC, USA; 2 Department of Clinical Sciences, College of Veterinary Medicine, North Carolina State University, 1060 William Moore Dr, Raleigh, NC 27607, USA

**Keywords:** Healthy dogs, CVBDs, Co-infection, Blood donors

## Abstract

**Background:**

Canine vector borne diseases (CVBDs) comprise illnesses caused by a spectrum of pathogens that are transmitted by arthropod vectors. Some dogs have persistent infections without apparent clinical, hematological or biochemical abnormalities, whereas other dogs develop acute illnesses, persistent subclinical infections, or chronic debilitating diseases. The primary objective of this study was to screen healthy dogs for serological and molecular evidence of regionally important CVBDs.

**Methods:**

Clinically healthy dogs (n = 118), comprising three different groups: Gp I blood donor candidates (n = 47), Gp II healthy dog volunteers (n = 50), and Gp III stray dogs (n = 21) were included in the study. Serum and ethylenediamine tetraacetic acid (EDTA) anti-coagulated blood specimens collected from each dog were tested for CVBD pathogens.

**Results:**

Of the 118 dogs tested, 97 (82%) dogs had been exposed to or were infected with one or more CVBD pathogens. By IFA testing, 9% of Gp I, 42% of Gp II and 19% of Gp III dogs were seroreactive to one or more CVBD pathogens. Using the SNAP 4DX® assay, Gp I dogs were seronegative for *Anaplasma* spp., *Ehrlichia* spp., and *B. burgdorferi* (Lyme disease) antibodies and *D. immitis* antigen. In Gp II, 8 dogs were *Ehrlichia* spp. seroreactive, 2 were infected with *D. immitis* and 1 was *B. burgdorferi* (Lyme disease) seroreactive. In Gp III, 6 dogs were infected with *D. immitis* and 4 were *Ehrlichia* spp. seroreactive. Using the BAPGM diagnostic platform, *Bartonella* DNA was PCR amplified and sequenced from 19% of Gp I, 20% of Gp II and 10% of Gp III dogs. Using PCR and DNA sequencing, 6% of Gps I and II and 19% of Gp III dogs were infected with other CVBD pathogens.

**Conclusion:**

The development and validation of specific diagnostic testing modalities has facilitated more accurate detection of CVBDs. Once identified, exposure to vectors should be limited and flea and tick prevention enforced.

## Background

Throughout the world, canine vector borne diseases (CVBDs) are caused by a group of widely distributed and regionally disparate arthropod borne pathogens. Dogs are considered competent reservoir hosts for several zoonotic vector borne bacteria and protozoa, and also serve as an important source of nutrition for many blood sucking arthropods, including fleas, mosquitoes, sand flies and ticks [1,2]. Importantly, some arthropods are competent vectors for transmission of more than one CVBD pathogen. Also, depending upon geographic location, acaracide use, life style, and other factors, dogs can be repeatedly exposed to the same or alternatively to multiple different vectors, ultimately resulting in sequential or concurrent infection with single or multiple CVBD pathogens [1-4]. Several factors contribute to the relatively high frequency of co-infections reported in dogs, as compared to other companion animals or humans from the same geographic region [1-4]. Collectively, these factors have contributed to growing medical interest regarding the impact of CVBDs on animal health and welfare, and have facilitated recent efforts to more thoroughly define public health implications associated with various CVBD pathogens.

Based upon a long evolutionary history and complex pathogen-vector-host interactions, persistent non-clinical or occult infection(s) are more prevalent among reservoir hosts, as compared to accidental hosts. Reservoir hosts tend to remain outwardly healthy without apparent clinical signs of illness, often despite concurrent, mild hematological, biochemical and urinalysis abnormalities. In contrast, accidental hosts more often develop disease manifestations that are accompanied by obvious pathophysiological abnormalities. Due to variable patterns of disease expression, ranging from subclinical to life-threatening infections, the diagnosis and medical management of occult CVBDs remains challenging. Historically, epidemiological screening and diagnostic assays were primarily based upon visualization of CVBD pathogens in patient blood smears and tissues, and/or serological assays that supported pathogen exposure, but by the nature of most assays (antibody detection) did not confirm active infection. Serology remains an important epidemiological modality to estimate CVBD prevalences among various dog populations and can also be used clinically to facilitate patient diagnosis. With the advent of highly sensitive and specific PCR assays, researchers and diagnosticians can confirm CVBD infections by amplification of organism-specific gene targets, followed by DNA sequencing or another molecular-based modality [1-3]. Therefore, recent improvements to molecular diagnostic techniques allow for more sensitive screening of non-ill dogs for occult infections, which facilitates more effective examination of zoonotic concerns and provides novel insights for the worldwide management and control of CVBDs. The purpose of this study was to determine the serological and molecular prevalence of CVBD pathogens in blood donor candidates, clinically healthy volunteer dogs, and stray dogs in North Carolina, USA.

## Methods

Serum and ethylenediamine tetraacetic acid (EDTA) anti-coagulated blood specimens were collected from 118 clinically healthy dogs, representing three different study groups. Gp I consisted of 47 dogs sampled between July 2009 and June 2011, that were being screened prior to acceptance as blood donors at the Veterinary Health Complex, North Carolina State University. As a component of the blood donor screening process, complete blood count and urinalysis results were available for Gp I dogs. Gp II consisted of 50 healthy dogs sampled between August 2012 and March 2013 belonging to veterinary students, technicians, faculty and local volunteers at the College of Veterinary Medicine, North Carolina State University who provided blood sample access to their dogs for Institutional Animal Care and Use Approved research studies (NCSU-IACUC 11-051-0). Prior to sampling, each owner of Gp II dogs signed an informed consent and completed a brief questionnaire. Prior to obtaining blood samples, these dogs were examined by a veterinarian and deemed healthy. Gp III consisted of 21 stray dogs that were sampled from 2nd February through 22nd March 2010 at a local animal control facility. All three dog groups were screened for regionally important CVBDs, as described below.

### Serology

Serum specimens were tested by immunofluorescent antibody (IFA) assays using *Bartonella vinsonii* subsp. *berkhoffii* (*Bvb*) genotypes I, II, III, *Bartonella henselae* (Houston 1 ITS genotype), *Bartonella henselae* (San Antonio 2 genotype), *Bartonella koehlerae*, *Ehrlichia cani*s, *Babesia canis*, and *Rickettsia rickettsii* as antigens. The sources of antigens for these IFA assays have been described previously [5,6]. Each serum sample was screened at dilutions of 1:16 to 1:64. All sera that were reactive at 1:64 were then further tested with two-fold dilutions out to 1:8192. A cutoff titer of 1:64 was used to define a seroreactive titer. All serum samples were also screened using a commercial ELISA-based kit (SNAP® 4DX®, IDEXX Laboratories Inc, Westbrook, ME) for *Dirofilaria immitis* antigen, and antibodies to *Anaplasma phagocytophilum*, *Ehrlichia canis* and *Borrelia burgdorferi* C6 peptide [7].

### *Bartonella* alpha proteobacteria growth medium (BAPGM) culture

*Bartonella* spp. BAPGM enrichment blood culture/polymerase chain reaction (PCR) was performed as previously described [8].

### Polymerase chain reaction and sequencing

*Bartonella* intergenic transcribed spacer (ITS) region was performed targeting the region between the *Bartonella* 16S - 23S ribosomal RNA genes. Primers and PCR conditions were previously described [5,6]. Similarly, primers and PCR conditions for *Babesia* spp, hemotropic *Mycoplasma*, *Rickettsia* spp, *Ehrlichia* and *Anaplasma* were used as previously described [9-11]. All PCR positive amplicons were sequenced and consensus sequences were aligned (Vector NTI Suite 10.1, Invitrogen Corp, CA, USA) with known sequences in GenBank using the basic local alignment search tool (BLAST) available from (http://www.ncbi.nlm.nih.gov/BLAST/). Previously described negative and positive controls were used for each PCR assay.

## Results

### Study animals

Gp I blood donor candidates included 28 (60%) male and 19 (40%) female dogs. The median age was 3 years (range– 10 months to 14 years). Sixteen breeds were represented, including Greyhound (8), Mixed breed (8), Terrier (7), German Shepherd (4), Laboratory Retriever (4), Golden Retriever (3), Australian Shepherd (2), Boxer (2), Siberian Husky (2), Belgian Malinois (1), Chow Chow (1), Doberman (1), English Setter (1), German Wirehaired Pointer (1), Great Dane (1), and Walker Hound (1).

Gp II healthy volunteer dogs included 29 (58%) male and 21 (42%) female dogs. The median age was 4 years (range – 3 months to 11 years). Seventeen breeds were represented, including Mixed breed (9), Laboratory Retriever (9), Terrier (7), Greyhound (4), Australian Shepherds (4), German Shepherd (4), Beagle (2), Maltese (2), Boxer (1), Bernese Mountain Dog (1), Border Collie (1), Corgi (1), Cocker Spaniel (1), German Wirehaired Pointer (1), Golden Retriever (1), Great Dane (1), and Mastiff (1). Based upon the questionnaire, 42 (84%) Gp II dogs were rescued and for the remaining 8 (16%) dogs the source of origin was not provided. Based on reported activities, forty (80%) were classified as indoor dogs, 8 (16%) were classified as indoor/outdoor and only 2 (4%) as outdoor only. Based on their primary residence, 32 dogs (64%) were from suburban areas, 10 (20%) were from rural areas and 8 (16%) from an urban environment. Flea and heartworm prophylaxis drugs were being administered to all Gp II dogs at the time of sampling and 47/50 (94%) dogs received a product for tick control. Based upon the vector exposure history, flea or tick infestations had occurred in 30 (60%) dogs. Gp III consisted of 13 (62%) male and 8 (38%) female dogs sampled at a local animal control facility of which 20% were surrendered by their owners and 80% were strays. Seven breeds were represented, including Mixed breed (10), Labrador Retriever (4), Walker Hound (2), Pit Bull Terrier (2), Golden Retriever (1), Australian Shepherd (1) and Beagle (1).

#### Complete blood count (CBC)

In this study, 70% of the screened blood donors with serological or molecular evidence to support CVBD exposure or infection had normal CBC values (data not shown), whereas the remaining dogs had subtle or non-specific hematological changes.

#### Serology

Cumulative serology results for the three groups of dogs are summarized in Table 1.

**Table 1:** Serological prevalence of canine vector borne pathogens

**Study group**	**Study population (N)**	**Overall IFA positivity N (%)**	**IFA antigens tested**									**No. of dogs exposed with > 1 IFA antigen**	**SNAP 4DX®**			
			***Bh*** **SA2**	***Bh*** **HI**	***Bvb*** **I**	***Bvb*** **II**	***Bvb*** **III**	***Bk***	***Rr***	***Ec***	***Bc***		**Lyme**	***E. canis***	***Anaplasma spp***	**Heartworm**
Group I	47	4 (9%)	0	0	0	0	0	0	1	1	0	2	0	0	0	0
Group II	50	21 (42%)	0	1	0	0	4	0	13	0	0	3	1	8	0	2
Group III	21	4 (19%)	0	0	0	0	0	1	0	1	0	2	0	4	0	6

**Legend:***Bk* – *B. koehlerae*; *Bh*SA2 – *Bartonella henselae* San Antonio 2; *Bh*HI– *Bartonella henselae* Houston 1; *Bvb* – *Bartonella vinsonii* subsp *berkhoffii* genotypes I, II, & III; IFA – Immunofluorescent antibody assay; *Rr* – *Rickettsia rickettsii*; *Ec* – *Ehrlichia canis*; *Bc* – *Babesia canis.*

##### Group I

By IFA testing, 4/47 (9%) blood donor candidate dogs were seroreactive to one or more CVBD pathogens. One dog each was *E. canis* or *R. rickettsii* seroreactive. Two dogs were seroreactive for more than 1 IFA antigen (one dog was *R. rickettsii* and *B. henselae* SA2 seroreactive and another dog was *R. rickettsii* and *Bvb* genotype II seroreactive). No dog was *B. canis*, *B. koehlerae*, *B. henselae* Houston 1, *Bvb* genotypes I and III seroreactive. SNAP 4DX® results for *Anaplasma* spp., *Ehrlichia* spp., and *B. burgdorferi* (Lyme disease) antibodies and *D. immitis* antigen were negative for all Gp I dogs (Table 1).

##### Group II

With IFA antibody titers greater than or equal to1:64 considered seroreactive, 21/50 (42%) healthy volunteer dogs were seroreactive to one or more CVBD antigen, of which 13/21 (62%) were only *R. rickettsii* seroreactive. Based on questionnaire data from the, 21 seroreactive dogs, 12 were rescues, 5 obtained from breeders, and the dog’s origin was not provided for the remaining 4 dogs. Ten of 13 *R. rickettsii* seroreactive dogs had tick or flea exposure histories. Three other dogs with histories of tick or flea exposure were seroreactive to more than one CVBD pathogen (2 dogs were *R. rickettsii* and *E. canis* seroreactive and one dog was *R. rickettsii* and *Bvb* genotype III seroreactive). All 4 *Bvb* genotype III seroreactive dogs had a history of flea and tick exposure. One dog was *B. henselae* Houston 1 seroreactive*.* No Gp II dog was *B. canis*, *B. henselae* SA2, *B. koehlerae, Bvb* genotypes I and II seroreactive by IFA testing. By SNAP 4DX®, 8 dogs were *Ehrlichia* spp. seroreactive of which 5 dogs had a history of tick or flea exposure. Seven of 8 *Ehrlichia* spp. seroreactive dogs reportedly received tick control prophylaxis. Two dogs were infected with *D. immitis* and 1 dog with a history of tick or flea exposure was *B. burgdorferi* (Lyme disease) seroreactive. No dog was *Anaplasma* spp. seroreactive (Table 1).

##### Group III

By IFA testing, 4/21 (19%) dogs sampled at a local animal shelter were seroreactive. One dog each was *B. koehlerae* or *E. canis* seroreactive*.* One dog was *B. henselae* SA2, *E. canis*, and *R. rickettsii* seroreactive. Another dog was *B. henselae* SA2 and *Bvb* genotypes, I, II and III seroreactive. By SNAP 4DX®, 4 dogs were *Ehrlichia* spp seroreactive and 6 dogs were infected with *D. immitis*. No dog was seroreactive to *Anaplasma* spp, *B. canis, B. burgdorferi* or *B. henselae* Houston 1 IFA antigens (Table 1).

#### *Bartonella* spp. BAPGM enrichment blood culture/PCR platform:

Cumulative BAPGM enrichment blood culture/PCR results for all three dog groups are summarized in Table 2.

**Table 2:** **Molecular prevalence of** ***Bartonella*** **spp and other canine vector borne pathogens**

**Study group**	**Study population (N)**	**Overall *Bartonella *** **spp prevalence N (%)**	**BAPGM diagnostic platform**			**Overall molecular prevalence of other CVBD pathogens N (%)**	**Hemotropic *Mycoplasma *** **16S PCR**	***Ehrlichia/Anaplasma *** **spp PCR**	**SFG *Rickettsia *** **PCR**	***Babesia *** **spp PCR**
			**Pre-enrichment PCR**	**Post-enrichment PCR**	**Isolate**					
Group I	47	9 (19%)	*Bk ***(3)**	*Bh *SA2 **(2)**	0	3 (6%)	*Mhc ***(3)**	0	0	0
			*Bh*HI **(1)**	*B*spp. **(2)**						
			*B*spp. **(1)**							
Group II	50	10 (20%)	*Bvb * III **(1)**	*Bvb * II **(1)**	*Bvb * II **(8)**	3 (6%)	*Mhc ***(1)**	*E. ewingii ***(2)**	0	0
Group III	21	2 (10%)	*Bvb * I **(1)**^**†**^	*Bvb* I (1)*	*Bvb * I (1)*	4 (19%)	*Mhc ***(1)**	*E. ewingii ***(2)**	0	0
			*Bvb * I (1)*				*C Mhp ***(1)**			

**Legend:***Bk* – *Bartonella koehlerae*; *Bh*SA2 – *Bartonella henselae* San Antonio 2; *Bh*HI– *Bartonella henselae* Houston 1; *Bvb* – *Bartonella vinsonii* subsp *berkhoffii* genotypes I, II, & III; CVBD – Canine vector borne disease; ^†^ - *Bvb* genotype I DNA sequence amplified from serum; * - *Bvb* genotype I DNA sequence amplified from blood; *Mhc* – *Mycoplasma hemocanis*; *C* Mhp- *Candidatus* M. hematoparvum*; B*spp – *Bartonella* spp; BAPGM – *Bartonella* Alpha Proteobacteria Growth Medium; PCR – Polymerase chain reaction; SFG – Spotted fever group.

##### Group I

*Bartonella* DNA was PCR amplified and sequenced from the blood of 9/47 (19%) blood donor candidate dogs. Prior to BAPGM enrichment blood culture, *B. koehlerae* DNA and *B. henselae* Houston 1 DNA were PCR amplified and sequenced from blood specimens from 3 and 1 dog, respectively. After BAPGM enrichment blood culture, *B. henselae* SA2 DNA was amplified and sequenced from two additional dogs. Because DNA sequencing of the 16S-23S ITS amplicon failed, the *Bartonella* sp. was not determined for three dogs (one dog was positive by pre-enrichment PCR and the remaining two dogs by post enrichment PCR). Subculture isolates were not obtained from any 7 or 14 day BAPGM enrichment cultures (Table 2).

##### Group II

*Bartonella* DNA was PCR amplified and successfully sequenced from the blood of 10/50 (20%) healthy volunteer dogs. Prior to BAPGM enrichment blood culture/PCR testing, only one dog was found to be infected with *Bvb* genotype III. After BAPGM enrichment blood culture, 9 dogs were infected with *Bvb* genotype II; however, isolates were obtained only from 8 dogs. (Table 2). Based upon questionnaire responses, 6/9 *Bvb* genotype II culture-positive dogs were either rescues or strays (3 each), whereas the remaining 3 dogs were obtained from a breeder. Four *Bvb* genotype II culture-positive dogs had a history of flea or tick exposure. Eight of 9 *Bvb* genotype II infected dogs reportedly received flea and tick control prophylaxis.

##### Group III

*Bartonella* DNA was PCR amplified and sequenced from 2/21 (10%) dogs from the local animal shelter. Prior to BAPGM enrichment blood culture, *Bvb* genotype I DNA was amplified and sequenced from the serum of one dog. After BAPGM enrichment blood culture, *Bvb* genotype I was amplified and sequenced from a second dog and subculture isolates were obtained from this dog’s 7 and 14 day BAPGM enrichment blood cultures (Table 2).

#### PCR and sequencing for other CVBD pathogens

Cumulative CVBD pathogen PCR results for all three groups of dogs are summarized in Table 2.

##### Group I

Three of 47 blood donor candidate dogs (6%) were *M. hemocanis* PCR positive. No dog was *Babesia* spp., *Rickettsia* spp, *Ehrlichia* spp. and *Anaplasma* spp. PCR positive (Table 2).

##### Group II

Three dogs (6%) were PCR positive for other CVBD pathogens, of which two dogs had a history of flea or tick exposure. *E. ewingii* DNA was amplified and sequenced from 2 (4%) dogs and *M. hemocanis* DNA was amplified from another dog. No dog was PCR positive for *Babesia* spp, *Rickettsia* spp. or *Anaplasma* spp. (Table 2).

##### Group III

Four dogs (19%) were PCR positive, of which 2 were infected with *E. ewingii* and one dog each was infected with *M. hemocanis* or *Candidatus* Mycoplasma hematoparvum (*C*Mhp). No dog was *Babesia* spp., *Rickettsia* spp. and *Anaplasma* spp. PCR positive (Table 2).

## Discussion

This study investigated the serological and molecular prevalence of regionally recognized CVBDs in blood donor candidates, clinically healthy volunteer dogs, and stray or surrendered dogs in central North Carolina. When serology and PCR results were combined, 97 (82%) dogs had been exposed to or were infected with at least one CVBD pathogen. Among Gps I, II and III, the distribution of dogs exposed to or infected with one or more CVBD pathogens was 34, 90 and 95%, respectively. Serologically, 42% of the 118 dogs were exposed to more than one CVBD pathogen. Based upon BAPGM enrichment blood culture/PCR, SNAP 4DX® heartworm antigen, and other CVBD PCR results, 33% of dogs in this study were actively infected with one or more than one CVBD pathogen. Despite being considered healthy by their owners or by the veterinarians obtaining blood samples for the volunteers and the dogs at the local animal shelter, the serological and molecular prevalence of CVBDs in Groups I and II were not substantially different from CVBD prevalences found in stray or surrendered dogs sampled at a local humane society. Stray or surrendered dogs were more often infected with *D. immitis* and exposed to *Ehrlichia* spp., reflecting frequent exposure to mosquitoes and ticks, and presumably failure to receive heartworm or tick preventive products. Compared to the other two groups, fewer (9%) blood donor candidate dogs were seroreactive to a CVBD pathogen, however, 12 (26%) of these dogs had occult infections with a *Bartonella* or hemotropic *Mycoplasma* spp. Because heartworm preventive products are routinely administered to most well cared for, client-owned dogs in North Carolina, no blood donor candidate was infected with *D. immitis*. Prior to expending money to test blood donor candidates for blood type, general health status and evidence of exposure to or infection with CVBDs, these dogs were qualitatively screened by blood bank personnel, potentially selecting against dogs with historical or frequent vector exposures. Group II dogs, belonging primarily to NCSU-CVM personnel or local volunteers were most often exposed to and/or infected with *Bartonella* spp.*, D. immitis, Ehrlichia* spp. and *Rickettsia* spp., most likely reflecting historical or more recent exposure to fleas, mosquitoes and ticks. It is possible that some rescued, local volunteer dogs had been successfully treated for heartworm disease (therefore heartworm antigen negative), as this possibility was not addressed in the brief questionnaire completed by the owners of these canine volunteers. As determined by questionnaire, veterinary students, technicians, faculty and local volunteers who provide access to their dogs for clinical studies often adopt rescued or stray dogs as personal pets. Based upon this study, pet-adopted, rescued dogs from central North Carolina often have historical CVBD exposures, which are similar to the vector exposure/infections found in stray and surrendered dogs. For CVBDs that induce long standing, occult bloodstream infections, stray and rescued dogs pose a potential risk if used as blood donors and could potentially bias laboratory reference ranges and “normal” reference data, if used as controls in research studies. Importantly, persistent occult CVBD infections would bias hematology, chemistry and clinical parameters when establishing laboratory reference ranges for “healthy” dogs. Dogs are considered biological hosts for most CVBDs, but they also serve as important environmental sentinels for determining the frequency and distribution of infected vector populations. For reasons that remain unclear, the majority of *Rickettsia* spp. seroreactive dogs in the southeastern United States are clinically healthy, despite the fact that *R. rickettsii* induces an acute, potentially life-threatening illness in dogs and human patients accompanied by a high fatality rate. The seroprevalence of rickettsial antibodies in dogs from endemic regions of the United States ranges between 26-60% respectively [12]. Following infection of dogs with *R. rickettsii* in North America, sterilizing immunity develops in conjunction with the acute febrile illness [13]. In contrast, dogs in Europe remain rickettsemic for at least one month when infected with *Rickettsii conorii* by *Rhipicephalus sanguineus,* confirming that dogs are competent reservoirs for *R. conorii*[14]. Seroreactivity to *R. rickettsii* antigens was found among all three groups; however, *Rickettsia* spp. DNA was not amplified from any clinically healthy dog in this study. There is extensive cross reactivity among spotted fever group rickettsiae; therefore, although *R. rickettsii* was used as the antigen source for IFA testing, it is not possible to infer which or how many spotted fever group *rickettsiae* these dogs might have been exposed to prior to specimen collection. Surprisingly, the majority of volunteer dogs (62%) were *R. rickettsii* seroreactive*,* compared to lower prevalences in Groups I (6.4%) and III (5%). As determined by questionnaire, the majority of Group II dogs were highly exposed to either ticks or fleas. Historically, tick transmission of *R. rickettsii* in the eastern United States has been attributed solely to *Dermacentor variabilis* and in the western United States to *D. andersoni*[15-18]. Subsequently, researchers at the Centers for Disease Control and Prevention (CDC; Atlanta, GA, USA) documented *R. rickettsii* transmission in the southwestern US by *Rhiphicephalus sanguineus* (commonly referred to as the brown dog tick or kennel tick) [17,18]. In this study, dogs that were *R. rickettsii* seroreactive were often *B. henselae* or *Bvb* seroreactive, suggesting exposure to a common vector or exposure to multiple vectors. As there does not appear to be cross reactivity between *Bartonella* and *Rickettsia* spp., these results may be attributed to simultaneous or sequential transmission of *Bartonella* and *Rickettsia* organisms to dogs by arthropod vectors [18,19]. A previous serosurvey study from our laboratory found a statistical association between seroreactivity to *B. henselae* and *R. rickettsii* antigens [20]. *B. henselae* is transmitted to cats and dogs [21] by *Ctenocephalides felis,* the common cat flea, which also transmits *Rickettsia felis*[22]. Therefore, it is possible that the serological association found in current and previous studies [21,22] reflects flea transmission of both organisms or alternatively independent exposure to both fleas and ticks, as *B. henselae* DNA has also been amplified from *Ixodes* spp. ticks in Europe [23] and *Ixodes pacificus* in North America [24].

*Bvb* genotypes I, II and III, *B. henselae* and *B. koehlerae* have been documented to infect both dogs and humans [25-30]. In this study, the overall *Bartonella* spp*.* IFA seroprevalence was 11% with the majority of seroreactive dogs found among the volunteer group. Among the six IFA antigens used in this study, *Bartonella* spp. seroprevalences ranged from 1 to 4%. In contrast, using the BAPGM diagnostic platform, the overall molecular prevalence of *Bartonella* spp. infection was 18%. Of the *Bartonella* spp. infected dogs, only 2% were seroreactive to the *Bartonella* sp. that was PCR amplified from their blood. A substantial number of *Bartonella* sp. bacteremic dogs do not have IFA antibodies [31]; which may reflect antibody negative (anergy) occult, host-adapted chronic infection, variation in antibody reactivity among strains of a *Bartonella* sp., or recently transmitted bacteremic infection that is documented prior to seroconversion. As these dogs were tested at only one point in time, it is not possible to determine if these *Bartonella* sp. infections were acute or chronic. In naturally-infected dogs, infection with *Bvb* is known to cause a prolonged bacteremia spanning months [32] and following experimental infection, *B. henselae* persisted for 94 days [33]. Interestingly, the *Bartonella* spp. detected in this study varied among groups, with an unexpectedly high prevalence of occult bacteremia (10-20%) documented in all three groups. Of those infected, *Bvb* genotype II was found in all but one Gp II dog, whereas only *Bvb* genotype I was found in Gp III, and neither genotype I or II was found in Group I. Previously, infection with *Bvb* genotypes I, II and III has been documented in sick dogs from the southeastern US; however, among sick dogs *B. henselae* was the predominant *Bartonella* sp. documented [34]. None of the 9 genotype II-infected Group II dogs were genotype II seroreactive. A recent study by Yore et al., found DNA of *Bvb* genotypes I and II in 11.3 and 6.3% of healthy dogs and flea pools in north central Florida, respectively [35]. The authors suggested that dogs may serve as a reservoir host for *Bvb* and fleas (both *C. felis* and *Pulex* sp.) may act as vectors for this pathogen. In pet dogs, both seroprevalence studies and blood culture isolation/PCR results indicate infrequent exposure to or active infection with any of the four *Bvb* genotypes; however, seroprevalence is higher in rural and working dogs, coyotes and feral dog populations [34,36]. As the 9 *Bvb* genotype II-infected dogs were sampled for BAPGM enrichment blood culture/PCR between February 14th and March 7th 2013, exposure to a common vector cannot be ruled out.

In this study the overall hemotropic *Mycoplasma* prevalence was 5%, with *M. hemocanis* and *Candidatus* M. hematoparvum prevalences of 4% and 1%, respectively. A previous study from our laboratory found an overall *M. hemocanis* and *Candidatus* M. hematoparvum prevalence of 1.3% among 506 dogs, with 0.6% of healthy and 0.8% of sick dogs infected [37]. Subsequently, Maggi et al. reported the prevalence of hemotropic mycoplasma infection was significantly greater in veterinarians, veterinary technicians, spouses of veterinary professionals, and others with extensive arthropod exposure and/or frequent animal contact when compared to patients with less frequent exposures [38]. Also, based upon recent studies, co-infections with *Bartonella* and hemotropic *Mycoplasma* spp. are commonly found in human patients [37-40]. In this study, 2 of 3 Gp I blood donor dogs were co-infected with *M. hemocanis* and a *Bartonella* spp. Thus, evolving evidence supports screening of canine blood donors for hemotropic *Mycoplasma* and *Bartonella* spp. co-infections and further supports the need to determine if co-infection with these organisms impacts the health of blood recipient dogs. However, our results cannot be extrapolated to all geographic regions or to all dog populations. Dogs that are adopted from environments that support exposure to heavy flea and tick infestation should be more intensively screened, before being used as blood donors. Additional serological and molecular studies are warranted to determine the prevalence of exposure and infection with CVBDs among diverse populations of clinically healthy dogs. In addition, greater research attention should focus on the potential medical importance of hemotropic *Mycoplasma* and *Bartonella* spp. co-infections in animal and human populations.

To prevent the risk of transfusion-associated infections with CVBD pathogens, blood donors should be screened prior to acceptance into a blood donor program. If not cost prohibitive, comprehensive screening of canine blood donors to optimally assess CVBD exposure and infection should include serology panels, PCR panels and the BAPGM enrichment blood culture/PCR platform. CVBD screening can be tailored to those pathogens documented to be endemic, based on geographic restrictions of disease, breed predilection, and documentation of disease transmission by transfusion [41]. These factors have been considered when making donor screening decisions for various blood donor programs. Once accepted as a donor, exposure to vectors should be limited; flea and tick prevention products should be used routinely; and annual screening should be performed on all canine blood donors.

## Conclusions

CVBD serologic and molecular assays should be used in combination to screen clinically healthy dogs being evaluated as potential blood donors or used as controls in biomedical research studies. To prevent future CVBD infections after initial screening, exposure to vectors should be strictly limited and acaracide products should be applied routinely and year round to blood donors and other healthy dogs.

## Abbreviations

(CVBD): Canine vector borne disease; (IFA): immunofluorescent antibody; (Bvb): *Bartonella vinsonii* subspecies *berkhoffii*; (BAPGM): *Bartonella* alpha Proteobacteria growth medium; (ITS): Intergenic spacer; (PCR): Polymerase chain reaction.

## Competing interests

In conjunction with Dr. Sushama Sontakke and North Carolina State University, Dr. Breitschwerdt holds U.S. Patent No. 7,115,385; Media and Methods for cultivation of microorganisms, which was issued October 3, 2006. He is the chief scientific officer for Galaxy Diagnostics, a company that provides diagnostic testing for the detection of *Bartonella* species infection in animals and human patients. All other authors have no potential conflicts.

## Authors’ contributions

NB performed the BAPGM enrichment blood culture/PCR testing and drafted the initial manuscript. SM, the veterinarian responsible for the NCSU Blood Donor screening program, contributed to the written content of the manuscript. MV is a veterinarian who performed physical examinations, sample collection and some of the CVBD testing reported in this study. JB performed IFA serological testing and abstracted data for the results section. EBB obtained funding for the study, edited initial and the final draft of the manuscript and oversaw the testing. All authors read and approved the final draft of the manuscript.
